# Advances in early detection methods for solid tumors

**DOI:** 10.3389/fgene.2023.1091223

**Published:** 2023-02-24

**Authors:** Bowen Jiang, Deqian Xie, Shijin Wang, Xiunan Li, Guangzhen Wu

**Affiliations:** Department of Urology, The First Affiliated Hospital of Dalian Medical University, Dalian, China

**Keywords:** tumor, early detection, CTC, cfDNA, EVs, liquid biopsy

## Abstract

During the last decade, non-invasive methods such as liquid biopsy have slowly replaced traditional imaging and invasive pathological methods used to diagnose and monitor cancer. Improvements in the available detection methods have enabled the early screening and diagnosis of solid tumors. In addition, advances in early detection methods have made the continuous monitoring of tumor progression using repeat sampling possible. Previously, the focus of liquid biopsy techniques included the following: 1) the isolation of circulating tumor cells, circulating tumor DNA, and extracellular tumor vesicles from solid tumor cells in the patient’s blood; in addition to 2) analyzing genomic and proteomic data contained within the isolates. Recently, there has been a rapid devolvement in the techniques used to isolate and analyze molecular markers. This rapid evolvement in detection techniques improves their accuracy, especially when few samples are available. In addition, there is a tremendous expansion in the acquisition of samples and targets for testing; solid tumors can be detected from blood and other body fluids. Test objects have also expanded from samples taken directly from cancer to include indirect objects affected in cancer development. Liquid biopsy technology has limitations. Even so, this detection technique is the key to a new phase of oncogenetics. This review aims to provide an overview of the current advances in liquid biopsy marker selection, isolation, and detection methods for solid tumors. The advantages and disadvantages of liquid biopsy technology will also be explored.

## 1 Introduction

With increases in human life expectancy, the incidence of tumors is increasing, and it has a major impact on public health worldwide (GBD, 2015 Risk Factors Collaborators, 2016). Malignant tumors deserve more attention than benign tumors in terms of the damage they cause and the medical costs they incur. The early detection of tumor especially premalignant lesions has led to higher cure rates, increased life expectancy, and lower medical costs. However, continued growth and metastasis of the tumor leads to a decline in the abovementioned benefits of detection. Until oncology drugs with high efficacies and lower side effects have been discovered, early detection and diagnosis of tumor especially premalignant is the most effective way to reduce mortality and prolong survival (Etzioni et al., 2003).

**FIGURE 1:**
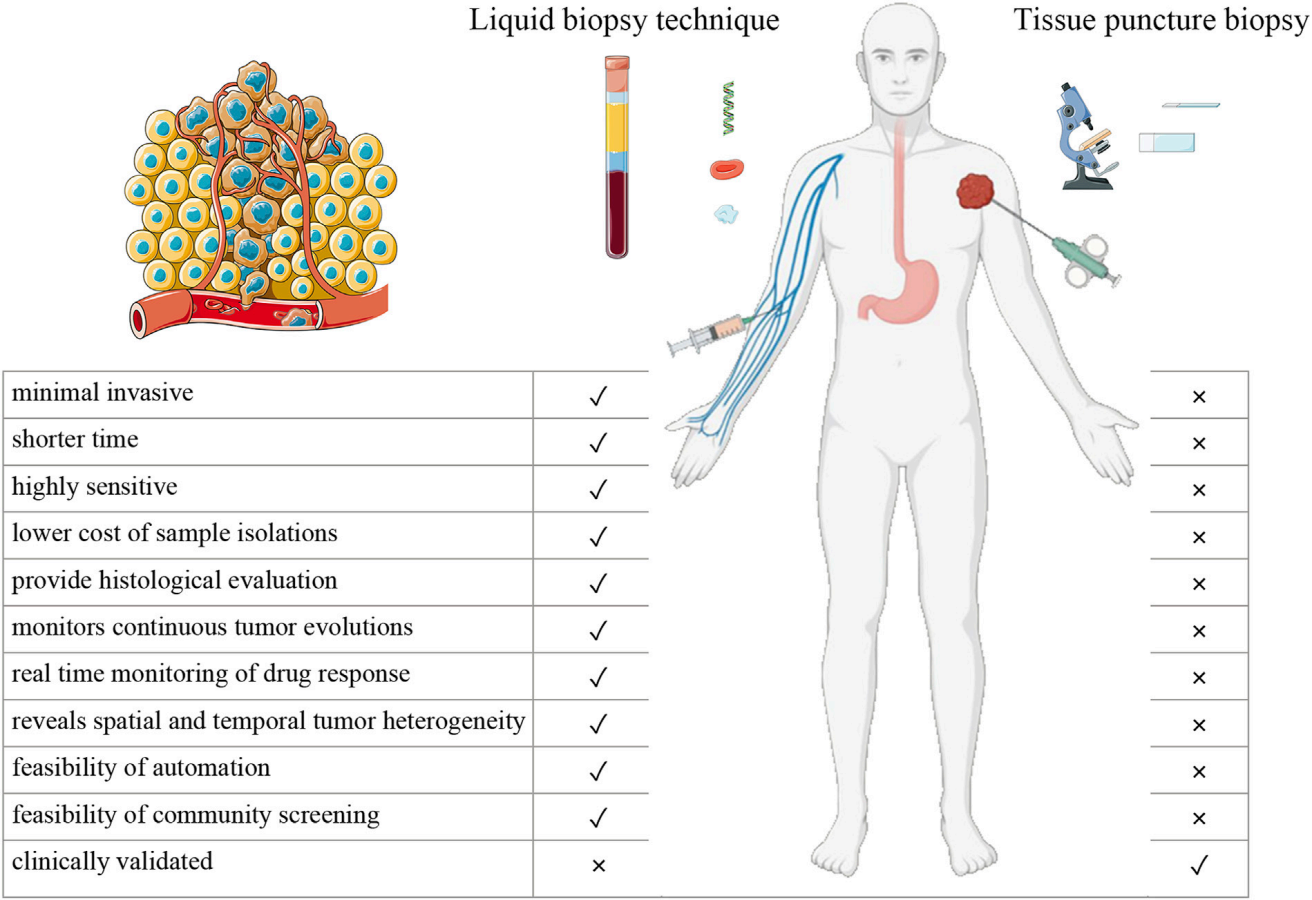
Differences between liquid biopsy technique and tissue puncture biopsy.

Traditional imaging and tissue biopsy techniques are the most widely used detection methods. However, in the early stages of tumor development, the information obtained from small samples is insufficient to diagnose tumors. Moreover, these detection methods have limited specificity for different tumors. Thus, multiple tests with considerable time costs are required for tumor diagnosis. Therefore, the widespread screening of healthy individuals is difficult. High financial costs associated with tumor diagnosis significantly burden public health (Figure 1). New methods for the early detection of solid tumors are urgently required to ease the burden on public health. With recent developments in genetics and proteomics, low-cost, more generalized, easy-to-use, low-injury, more sensitive, and specific solid tumor early detection methods are gradually becoming available. In this study, we reviewed the progress of research on tumor-based early detection methods.

## 2 Detection of tumors

Tumor detection begins with discovering the difference between tumors and normal cellular tissue, thus finding methods that can accurately and quickly differentiate them. The differences between tumor cells and differentiated physiological human tissue cells can be broadly classified based on the following: 1) physical differences (differences in cell size and morphology, as well as differences in tissue morphology and structure) and 2) biological differences (differences in growth and metabolism due to genetic changes). Therefore, detection methods are based on physical differences in tumor detection and biological differences in tumor detection.

Due to the physical differences in tumors, various imaging modalities and tissue biopsies are often needed to identify the tumor’s original site, in addition to sites of distant spread. In contrast, biological characteristics (from genes to proteins to differences in cell behavior) can be determined by indirect evidence of tumor presence and the type and direct evidence. An increase in immune cells associated with tumor formation is an example of indirect evidence that can be used to diagnose tumor growth. In addition, other cellular and metabolic changes caused by the tumor are detected such as an increase in CEA values. Indirect evidence can indirectly determine the presence of a tumor without the need to find direct evidence using methods such as puncture biopsy to locate a tumor that may not be present at high cost. Therefore, tumor detection methods based on biological differences are advantageous.

## 3 Biological characteristics of tumor cells

Differentiated physiological human tissue cells are programmed to undergo apoptosis eventually. Human tumor cells are distinguished from differentiated physiological human tissue cells by their uncontrolled and unlimited proliferation, invasion, and metastasis. Biological features are implicit in tumor cell formation. Tumor cells are genetically altered. These genetic alterations result in uncontrolled growth, metabolic changes, and angiogenesis in tumor cells.

These genetic alterations can be divided into three levels: 1) altered genetic material, 2) altered metabolites, and 3) altered cellular behavior.

### 3.1 Alterations in genetic material

Carcinogenesis is the alteration of genetic material within the cell that leads to tumor formation. Human genes that contain alterations in their genetic material are termed oncogenes. However, the abnormal expression of oncogenes in cells leads to carcinogenesis. For example, oncogene activation (KRAS and MYC), oncogene inactivation (APC), mismatch repair gene inactivation (PMS1), and gene overexpression (PTGS2) have been observed in colon cancer progression. Altered genetic material is the initiating factor for carcinogenesis.

### 3.2 Alterations in metabolites

Alterations in genetic material inevitably result in alterations in metabolites. The most prevalent alterations occur in genes corresponding to transcribed RNA products and encoded proteins. Thus, the levels of transcribed RNA products and encoded proteins are increased compared to normal levels in tumors. In addition, tumor cells secrete extracellular vesicles containing bioactive substances, and the interpretation of these intercellular messages varies.

### 3.3 Alterations in cellular behavior

The reduced surface viscosity of tumor cells enables them to be aggressively metastatic. This leads to the increased motility of tumor cells in body fluids and their metastasis body. As a result, tumor cells may be detected throughout the body, especially in body fluids. This also accounts for metastasis observed in malignant tumors.

## 4 Liquid biopsy technique

**FIGURE 2:**
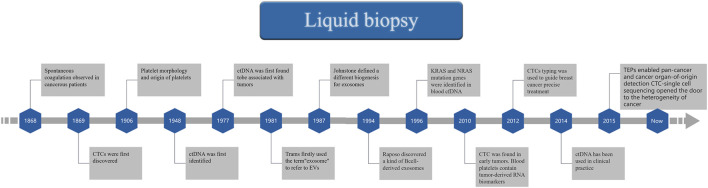
A Timeline of Liquid biopsy.

The liquid biopsy technique is used to analyze tumor-related substances (such as cells, nucleic acids, proteins, and other metabolites) in blood or other body fluid samples in a non-invasive or minimally invasive manner. Compared with traditional tissue biopsy, it enables the detection of indirect and direct information confirming a tumor’s presence. This technique is more convenient and flexible for patients since it is a diverse testing modality that is less invasive and utilizes detection targets that are more sensitive than traditional methods (Figure 2). The liquid biopsy technique has developed rapidly and achieved remarkable results in recent years as a detection method with great advantages and have made many advances in the field of breast cancer, colon cancer, lung cancer, melanoma and many other cancers (Pinzani et al., 2021). In addition to improving the accuracy of the assay and finding more new markers, the variety of body fluid samples obtained has been expanded in recent years. Test objects have also expanded from samples taken directly from cancer to include indirect objects affected in cancer development.

### 4.1 DNA

The earliest change that transforms differentiated physiological human tissue cells into tumor cells is a change in genetic material. Thus, detecting alterations in genetic material early is advantageous. Body fluids, particularly blood, contain a variety of biological information. For example, cell-free DNA (cfDNA) is significantly altered in patients with cancer, and analysis of blood enables early detection of tumor cells. In addition to cancer, alterations in physiological states, such as onset of inflammation or by exercising, lead to an increase in the quantitative level of cfDNA; this elevated concentration is not always indicative of malignancy (Atamaniuk et al., 2004). Thus, research needs to be focused on circulating tumor cell DNA (ctDNA), which accounts for only 0.1%–10% of the total circulating cell-free DNA (cfDNA), but has a higher sensitivity.

The sensitivity of cfDNA is well established. As a consequence, over 50 million colon cancer cells were accurately detected by analyzing ctDNA. Even so, imaging localization puncture biopsy is unable to detect the same volume of solid tumors (Diaz et al., 2012). CfDNA was first described 70 years ago and has since been widely used to detect tumor cells (Mandel and Metais, 1948). With the rapid development of next-generation sequencing (NGS) technology, ctDNA sequencing has enabled routine clinical diagnosis of tumors in their early stages with higher sensitivity than a tissue biopsy. In addition, cfDNA isolation is not labor-intensive; rather it can be almost entirely automated (Kahlert, 2019). This is a major advantage of its usage in the clinic setting. cfDNA analysis has two applications: 1) it is quite extensive and enables a broad range of research through genomic analysis and sequencing; and 2) it contains specific genomic regions where specific mutations can be identified and studied to improve the accuracy of detection techniques, including those used in tumor detection. For example, droplet digital polymerase chain reaction is a commonly used method for cfDNA assays (Holm et al., 2020). Recent developments in NGS have also ensured that NGS-based assays are capable of detecting cfDNA with greater sensitivity in large regions of the genome.

However, there are still obstacles in the clinical application of using cfDNA in early-stage tumor detection. First, cfDNA concentrations in patients with asymptomatic early-stage tumors are extremely low in some tumors, especially situ premalignant lesions (Roy and Tiirikainen, 2020). Thus, to improve sensitivity, a large number of blood samples are required (Sant et al., 2022). cfDNA produced by normal somatic cells and hematopoietic stem cells, can interfere with identification of cfDNA produced by tumor cells (Atamaniuk et al., 2004; Chen and Zhao, 2019; Song et al., 2022). Thus, the detection sensitivity of cfDNA depends on the signal-to-noise ratio (Kahlert, 2019). Many factors can affect the sensitivity of the assay, such as the induction of direct tumor rupture prior to blood collection and cell membrane lysis after blood collection. Recent studies have provided new ideas for reducing this interference. ctDNA differs from other cfDNA and exosomal DNA by the length of its base pairs. ctDNA fragments were previously shown to have 20–50 base pairs in cancer patients. These ctDNA fragments are relatively smaller than cfDNA (Underhill et al., 2016). Exosomal DNA, another interfering factor, was found to be mostly double-stranded and composed of larger nucleotide fragments than cfDNA and ranged from 2.5 to 10 kB in length (Thakur et al., 2014).

The low sensitivity of sequencing (Moding et al., 2021) can be solved through the detection of DNA methylation. For example, this method has been clinically used to validate the effects of PTGER4/SHOX2 genes in lung cancer, as well as the GSTP1 and GSTP1 genes in prostate cancer (Roy and Tiirikainen, 2020; Luo et al., 2021).

Second, most genetic mutations that result in cancer are not specific to a single type of tumor. Therefore identifying the cell in which the genetic mutation occurred may be difficult. The clinical significance of positively identifying the cell in which the genetic alterations occurred is yet to be elucidated. However, this might be useful in slowing the transformation of the mutated cells into cancerous cells, and it may also be possible that the transformation may be completely inhibited as well (García-Pardo et al., 2022).

In conclusion, cfDNA testing has a very promising future. Furthermore, its importance has been established in other clinical applications, such as in the molecular genotyping of advanced disease and the detection of acquired drug resistance (Chae and Oh, 2019). In addition, its role is widely recognized by the European Society of Medical Oncology (ESMO) and other organizations (Pascual et al., 2022). However, the use of this technology in early tumor detection needs further research. For cfDNA to become the gold standard for tumor detection in clinical practice, standardization of its separation and analysis is required.

### 4.2 RNA

RNA is another type of genetic material that can be obtained by liquid biopsy and used as a biomarker. Circular RNA (circRNAs), discovered in 1970, are a class of non-coding RNA that are produced mainly by pre-mRNA splicing (Hsu and Coca-Prados, 1979). The rapid development of high-throughput transcriptome analysis technologies in recent years has made it possible to detect circRNAs in body fluids. Studies have found stable and significant differences in the type and content of the splicing byproduct circRNA, between cancer patients and healthy controls; although previously thought to have no biological significance (Dragomir and Calin, 2018; de Fraipont et al., 2019). This suggests that the presence of circRNAs in body fluids may serve as novel biomarkers for early cancer screening and monitoring. As of May 2020, 112 differentially expressed circRNAs associated with a dozen cancers were visible in PubMed (Wang S. et al., 2021). Recently, related studies have also been published. For example, a group of 8-circRNAs was shown to be a potential diagnostic biomarker for the early detection of gastric cancer (Roy et al., 2022). In addition, studies on other RNAs, such as miRNAs, in early cancer screening have yielded some results. However, the advantages and difficulties encountered in their practical implementation are similar regardless of the type of RNA (Valihrach et al., 2020; Ding et al., 2022).

Theoretically, RNA as a transcription product should be more abundant than DNA fragments in body fluids and more stable based on the covalently closed continuous loop structure of circRNA (Li et al., 2015). It is not easily degraded by nucleic acid endonucleases, is expressed in a stage-specific manner, and is abundant not only in tissues and cells but also in body fluids (Arnaiz et al., 2019). This demonstrates its great potential as a marker for the early screening and detection of cancer progression.

However, it has to be acknowledged that there are great difficulties and challenges in the clinical application of RNA. The first issue is their abundance and the inaccuracies in their detection of body fluids (Wang S. et al., 2021). RNA-Seq and the gold standard RT-qPCR techniques can solve this issue (Hong et al., 2020). Even so, these techniques are associated with higher costs, complex analytical processes, and large workloads (Valihrach et al., 2020). Moreover, research on RNA as a cancer biomarker is limited. Furthermore, information on its role in tumorigenesis and tumor progression is also limited, and the results rely heavily on algorithmic models of machine learning. Therefore, its clinical application still has a long way to go. However, circRNAs are still efficacious when combined with traditional cancer biomarkers as supporting evidence (Qiao et al., 2019). In addition, it can also be combined with imaging techniques to improve accuracy. Imaging has recently seen tremendous progress and similar research ideas in the direction of tumor detection. Radiomics is one of them. It links imaging and oncology and uses machine learning methods to build models to improve the accuracy of detection (Liu Z. et al., 2019). Similar to RNA detection, further research also focus on machine learning related priorities such as interpretability. Thus, we expect that they can be combined to improve the accuracy of diagnosis.

### 4.3 Protein

**FIGURE 3:**
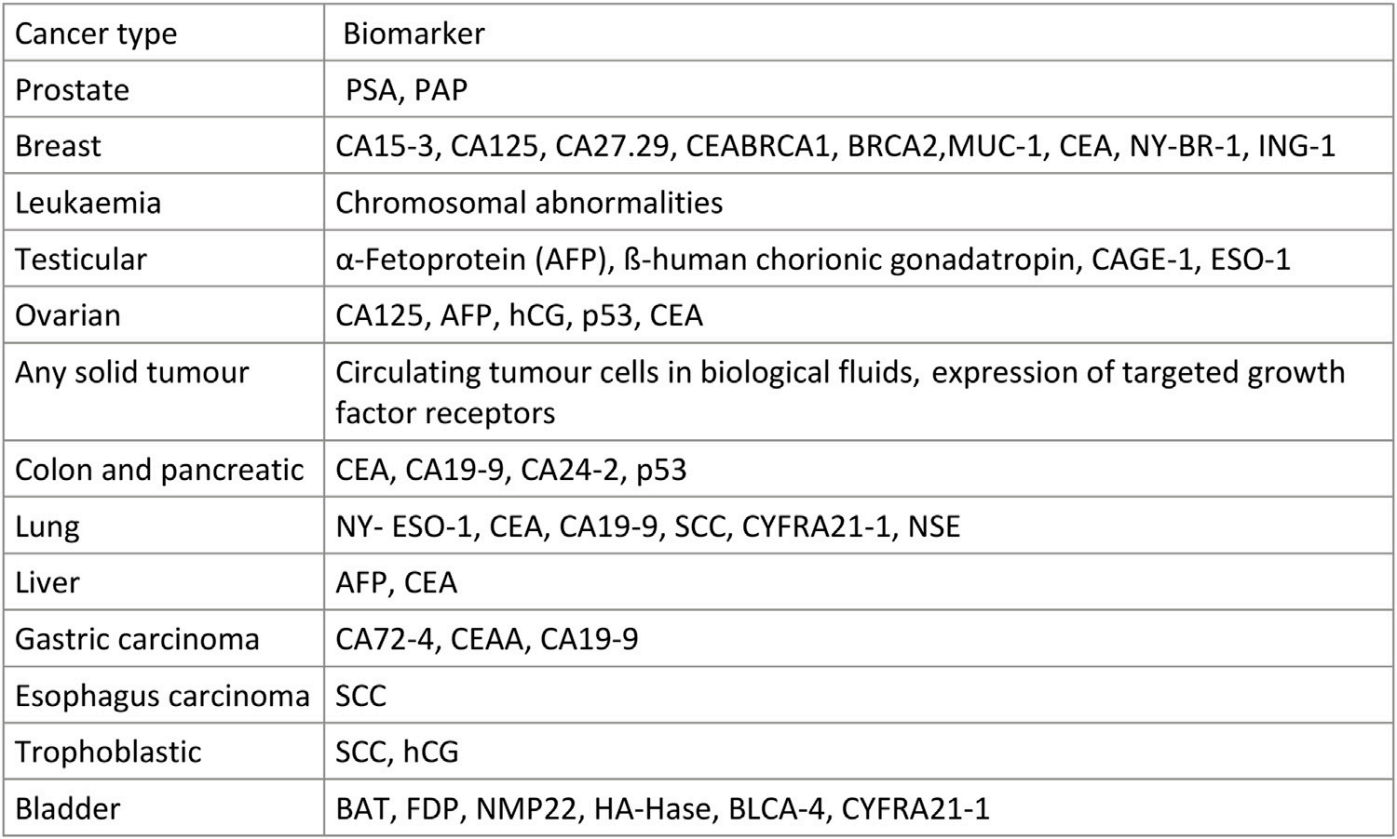
Some common Tumor biomarkers.

Numerous studies have shown that some proteins are expressed at higher levels in tumor tissues than in normal tissues. Therefore, these protein biomarkers have been used in clinical practice for many years as important tools for the diagnosis of tumor diseases. The basic principle of this assay is the specific binding of a tumor-specific antigen to its corresponding antibody. The markers used clinically for tumor diagnosis and the prognosis is listed in (Figure 3) (Jayanthi et al., 2017) The thresholds for these commonly used tumor markers have increased, and they have become stable and effective diagnostic aids. Moreover, protein biomarkers have made new discoveries in detection methods and the search for new markers and methods.

For example, plasma heat shock protein 90 alpha has been used as a pan-cancer biomarker for diagnosis (Liu W. et al., 2019). TREM2 is used to determine prognosis (Cheng et al., 2021). There are also studies aimed at changing the methodologies of enzyme-linked immunosorbent assay (ELISA)-based biomarker assays, which include disadvantages such as being slow and expensive reagents that are unable to detect multiple biomarkers simultaneously. This was the basis for the development of electrochemical, optical, and mass-based biosensor research (Jayanthi et al., 2017).

The main reasons for the bottleneck observed in the development stages of biosensors include the need to have increased sensitivity, selectivity, real-time analysis, and cost-effectiveness. Unlike in the laboratory setting, clinical applications require the need to be stable. Thus, biosensors are not yet ideal for clinical applications. New advances in proteomics and genomics are needed to further our understanding of the highly complex mechanisms of cancer cells (Jayanthi et al., 2017).

### 4.4 Extracellular Vesicles

Extracellular vesicles (EVs) are lipid bilayer particles released from cells, ranging from 30 to 5,000 nm in diameter. EVs can be divided into exosomes, microvesicles, and apoptotic vesicles. Among them are exosomes, a class of endosome-derived extracellular vesicles ranging in size from 30–100 nm, (Li et al., 2022), and microvesicles derived from the plasma membrane, which range from 50 to 1,000 nm but may be larger (up to 10 µm) (van Niel et al., 2018). EVs have lipid bilayers, are released by almost all cells, are rich in exosome-associated proteins and other bioactive molecules, and transmit important information between cells (Meldolesi, 2018). After obtaining clinical specimens and enriching, purifying, and isolating them, the protein and nucleic acid information contained in tumor-derived extracellular vesicles can be used as diagnostic markers for a variety of cancers. For example, EVs of CD63 and caveolin-1 can be used as potential markers for melanoma, (Logozzi et al., 2009), and metastasis suppressor 1 can be a predictive marker for liver metastasis in pancreatic cancer patients (Costa-Silva et al., 2015). There are studies in which EVs are used for early tumor detection; for example, an increasing number of cell surface proteoglycans glypican-1, (Melo et al., 2015), and KRAS mutations can be used as adjuncts for early detection of pancreatic cancers (Melo et al., 2015).

Exosomes contain DNA and RNA from parental cells. These DNA and RNA contained in exosomes can provide effective information for the diagnosis and treatment of patients with various types of cancer. Exosomal DNA was recently discovered. However, evidence supporting its existence was limited until Balaj discovered the existence of single-stranded DNA (ssDNA) (Balaj et al., 2011). However, it is now widely accepted that exosomes contain DNA (Allenson et al., 2017) along with RNA (Wang et al., 2020). Exosomal miRNAs such as miR-1246, miR-4644, miR-3976, and miR-4306 are highly sensitive biomarkers in pancreatic cancer patients (Madhavan et al., 2015). Elevated levels of miR-193a-3p, miR-210-3p, and miR-5100 in exosomes are non-invasive biomarkers of cancer progression in non-small cell lung cancer (Zhang et al., 2019). In addition, circRNAs are found in the free form in the blood and exosomes. Elevated concentrations of specific circRNAs in exosomes correlate with the presence of ovarian, prostate, and lung cancers (Wang Y. et al., 2019). In addition to miRNAs and circRNAs, other non-coding RNAs, such as lncRNAs in exosomes also play an important role in the diagnosis and monitoring of tumors (Wu et al., 2020). Recent studies have suggested that exosomal lncRNA-GC1 can be used as non-invasive biomarkers in the early detection of gastric cancer and in monitoring its progression (Guo et al., 2020).

In contrast to previously described limitations in detection sensitivity and signal-to-noise ratio of cfDNA and RNA, exosome-based assays may have theoretical advantages. The double-lipid outer membrane protects the encapsulated components from degradation and prevents their destruction during freeze-thaw and transport oscillations (Kahlert and Kalluri, 2013). In some cancers, exosome-based assays are more advantageous than cfDNA sequencing. For example, in patients with advanced or metastatic pancreatic cancer, KRAS mutations in exosomal DNA are detected at a much higher rate than cfDNA (Allenson et al., 2017). In addition, recent experiments have shown that EVs are more effective than whole serum samples in detecting CNS tumors (Dobra et al., 2020).

The main problem in the clinical translation of this assay is the difficulty in extracting the EV contents of the extracellular vesicles of tumor cells. The most common extraction method today is ultracentrifugation, which involves manual labor rather than full automation. Even so, this extraction can be achieved with biomarkers such as cfDNA (Kahlert, 2019). Another physical-scale separation method is ultrafiltration, which is limited in the manual steps required to complete it, but also requires the use of the squeeze filtration enrichment method for its completion. The latter method causes vesicles to rupture and affects the results of its subsequent analysis (Batrakova and Kim, 2015). The two methods can also be used in combination to provide an advantage in microbubble enrichment and to identify biomarkers that can be used to determine disease prognosis (Rood et al., 2010).

Immunoaffinity-based detection and separation methods include the magnetic immunocapture of antibody-coated magnetic particles that has an increased efficacy compared to using centrifugation for enrichment (Zarovni et al., 2015). Related kits, such as ExoQuick PLUS, ThermoFisher, CUSABIO, iZON, qEVSingle, and 101Bio, may also be used in EV separation of blood samples (Mathivanan et al., 2010). The scarcity of the final target product is a limitation of liquid biopsies that needs further exploration. In addition, the non-selective processing of similar biomarkers in a specimen that includes body fluids poses the potential risk of masking information or generating false positive results (Kahlert, 2019). Thus, the clinical translation of EVs assays for early tumor diagnosis depends on further confirmation of relevant studies.

### 4.5 Circulating tumor cells

Circulating tumor cells (CTC) were found in the peripheral blood of patients in the 19th century. CTCs are released from the primary tumor into the circulatory system, which explains the distant metastasis of the tumor *in vivo*. CTC assays soon achieved significant results in various aspects of disease detection and have been translated into clinical practice after a long period of validation (Lin et al., 2021). CTCs have proven to be a great success in detecting tumors and monitoring progression, not only because of the ease of sampling due to its usage of the liquid biopsy method but also because it provides a visual and dynamic picture of tumor progression in real-time, independent of metabolic and other complex factors. This makes it more accurate and convincing than other biomarkers detected in the blood.

Analyses of CTC, therefore, provide a clear picture of tumor progression. In addition, the CTC count is a strong indicator of treatment efficacy. For example, studies have shown that treatment efficacy in patients with breast cancer is positively correlated with the degree of reduction in CTC count (Smerage et al., 2014). Additional information about the tumor can now be obtained from CTCs isolated from blood samples using isolation techniques.

The various methods used to detect CTC from samples are broadly classified into three categories: 1) enrichment of CTC based on the immunoaffinity of cell surface molecular markers, 2) isolated enrichment based on the physical properties of CTC, and 3) direct analytical detection without enrichment.

Immunoaffinity based on cell surface molecular markers to enrich CTC uses antibodies against cell surface markers attached to the device surface or magnetic material. This method is subdivided into two subcategories: 1) positive and 2) negative enrichment (according to whether reverse screening is performed against CTCs or background cells). There are many molecular markers on the surface of CTC, and the most common is EpCAM, a common CTC marker for cancers of epithelial origin (Gires et al., 2020). EpCAM expression varies between cancer types, and strong EpCAM expression can be used for CTC detection in some cancers. For example, breast and prostate cancers have strong EpCAM-positive CTCs and have been shown to have clinical value as prognostic markers (Gorin et al., 2017; Fabisiewicz et al., 2020). Other cancers of epithelial origin, including hepatocellular carcinoma, (Xia et al., 2021), pancreatic cancer, (Varillas et al., 2019), and colorectal cancer (Marcuello et al., 2019) also have a high rate of EpCAM-positive CTC. Thus, CTC information can be used to determine the prognosis and survival of patients with distant metastases. There are many methods for detecting EpCAM. The most common method for detecting EpCAM is EPISPOT analysis using antibody fixation, culture, or amplification of membrane nodules of epithelial cell adhesion molecules such as EpCAM. Even so, the only FDA-approved CTC diagnostic system, the CellSearch™ System, is an immunomagnetic separation system that uses ferromagnetic fluid beads attached to EpCAM antibodies (Allard et al., 2004; Cohen et al., 2008).

There are CTCs that cannot be used as valid biomarkers of EpCAM positivity. For example, some tumors are inherently EpCAM-negative or have low EpCAM expression. Furthermore, reports have shown that tumors undergo an epithelial-to-mesothelial transition (EMT) after entering the circulatory system. This results in an increased number of negative CTCs compared to EpCAM-positive CTCs. Therefore, EpCAM is not a widely used biomarker. Other markers, such as SNAIL, TWIST, and EMT-related transcription factors, such as the ZEB family, assist in screening CTCs (De Craene and Berx, 2013; Mittal, 2018). For example, in breast cancer, the use of dual markers to indicate baseline EpCAM and N-calmodulin can correct CTC isolation and identification of single markers (Wang Z. et al., 2019; Wang et al., 2021 Z.). However, most EMT-related biomarkers are cytoplasmic or nuclear proteins. Therefore, they cannot be isolated using assays based on molecular markers on the cell membrane surface. Thus, targeting stem cell-related markers such as, CD133; mesenchymal markers, such as vimentin; and other biomarkers, including native tumor-related markers, such as human epidermal growth factor receptor 2 (HER2), (Nanou et al., 2020), estrogen receptor, (Forsare et al., 2020), folate receptor, (Chen et al., 2020), survivin, (Cao et al., 2011), prostate-specific membrane antigen, (Yin et al., 2018), and human high molecular weight melanoma-associated antigen (HMW-MAA) (Lucci et al., 2020) were developed for mixed cross-use to improve the detection of CTCs with extremely heterogeneous antigens. Another method of negative enrichment is to design an enrichment method based on the background cells to be excluded for CTC collection rather than collecting CTCs with heterogeneity. In addition to some professional and mature commercial platforms, such as the EasySep Depletion Kit, (Yang et al., 2009), technologies for positive enrichment, such as MACS and CTC-iChip, can actually be used to simply replace EpCAM with CD45 for negative enrichment (Ferreira et al., 2016). Exclusion of non-CTC cells by their major antigenic markers, including CD45/CD66b (granulocytes), CD235a (erythrocytes), CD41/CD61 (platelets), CD4/CD8 (lymphocytes), CD11b/CD14 (macrophages), and CD34 (hematopoietic progenitor cells/endothelial cells), could improve the sensitivity of CTC enrichment. Even so, single negative enrichment was not possible due to the crossover of antigenic markers and CTC exclusion. Furthermore, limitations in CTC crossover and incomplete exclusion decrease its specificity (Lara et al., 2006; Yang et al., 2009).

Physical properties can be used to separate the enrichment based on differences in diameter size, physical plasticity, and the dielectric mobility of CTCs relative to blood cells. These differences can be used for membrane filtration, microfluidic channels, density gradient stratification, inertial focusing, and dielectric mobility methods. In addition, the separation of CTC is detected by the significant difference in the physical plasticity of WBC and CTCs (Shaw Bagnall et al., 2015). For the difference in diameter size, CTCs (mean diameter −15.6 μm) and WBCs (diameter range 7–15 μm) (Dolfi et al., 2013) were less evident than physical plasticity, so it is understood that artificial means can be used to artificially increase the diameter contrast between the two types of cells using microbeads with specific antigen antibodies, such as anti-EpCAM antibodies. The latter results in increased recovery and purity of the cells (Kim et al., 2012). However, this undermines the significant advantage of using physical properties to separate the enrichment, as the enriched selected CTCs have no antibodies on their surfaces and are thus more susceptible to further processing.

The third method, the enrichment-free method, was developed to avoid the loss of time, labor, and sample accuracy associated with the first two methods. Advances in high-throughput single-cell imaging have made it possible to identify CTCs in blood samples without enrichment directly (Han et al., 2016). For example, the principle of using Imaging flow cytometry to separate CTCs from WBCs using physical parameters such as differences in diameter sizes and karyoplasmicratio ratios of the 2 cell types (Kleiber et al., 2021). Another method, photoacoustic flow cytometry (PAFC), can accomplish laser-based CTC detection in real-time. A technique combining multicolor high-speed photoacoustic microscopy and microfluidics for cell imaging, photoacoustic imaging flow cytometry (PAIFC), has also been developed to overcome the problems associated with the need to pre-process blood samples with excellent sensitivity and specificity (Jin et al., 2021).

CTCs can provide critical information about tumor characteristics, predict treatment response outcomes at early detection and provide direct evidence of epigenetic changes in tumor-associated genes in real-time during treatment. Most Importantly, its close association with distant tumor metastasis plays a key role in determining the choice of protocols for clinical treatment by patients and physicians. In addition, CTCs can also help model tumors. For example, the instability of the tumor genome was monitored by genetic analysis of CTCs. It is particularly helpful in assessing treatment outcomes and precision medicine in terms of tumor drug resistance and metastasis. It is also possible to analyze the CTC transcriptome using single-cell sequencing technology, which has rapidly developed in recent years, (Lim et al., 2019; Dong et al., 2020), to improve diagnostic tools and perform *in vivo* and *in vitro* drug treatment trials. However, the current clinical application of CTC still depends on the analysis of traditional CTC cell counts and molecular phenotypes. A more comprehensive characterization of CTC based on genome, transcriptome, and proteome, as well as high-throughput sequencing, will further facilitate the clinical application of CTC detection. However, limited techniques for studying single cells and difficulties in data analysis make them, especially proteomes, unavailable for widespread clinical use.

### 4.6 Non-blood Samples

The previously discussed liquid biopsy techniques use blood samples. However, liquid biopsies are not only performed on blood samples (plasma and serum). Many studies have shown that other body fluids, such as cerebrospinal fluid, saliva, urine, and semen (Rzhevskiy et al., 2022) can be used in liquid biopsies (Street et al., 2012). Using body fluids other than blood can be advantageous. For example, body fluids, such as saliva and urine, are more accessible, non-invasive, and inexpensive. For detecting tumors in these regions, such as oral cancer, saliva has a significant advantage over samples of blood (Lousada-Fernandez et al., 2018).

Recently there have rapid advances in saliva sample-based diagnostic techniques used in the early detection and progression monitoring of cancers. For example, improvements are currently being made to the following techniques: 1) the use of electric field-induced release and measurement (EFIRM) (Tu et al., 2020) to detect EGFR mutations (tyrosine kinase structural domains) in body fluids such as the saliva of patients with non-small cell lung cancer (NSCLC) (Li et al., 2020); 2) the exploration of salivary biomarkers, such as Foxp1 and Gng2 mechanisms in pancreatic cancer; (Lau et al., 2013); and 3) the exploration of non-genomic based markers, such as the spectroscopic analysis of salivary metabolites and changes in the oral microbiota. Studies have shown that both elevated porphyrin levels (Yuvaraj et al., 2014) and changes in the oral microbiota (Deo and Deshmukh, 2020) are associated with oral squamous cell carcinoma and can be used as a potential adjunct in the detection of tumors.

Similarly, liquid biopsies based on urine sampling are additional methods for detecting cancer cells. This technique is based on the fact that abnormal cells and most biomolecules secreted by tumors are likely to enter the urine through the urinary system. Thus, this technique is uniquely advantageous since it enables repeated serial sampling that can be used to monitor cancer progression or recurrence. In addition, urological tumors, such as bladder cancers (Togneri et al., 2016; Zhang et al., 2021) and prostate cancers, can be used in the detection of colorectal cancer (Zhou et al., 2022).

The main biomarkers used for cancer detection in urine samples are exfoliated bladder cancer cells (EEBC), cell-free DNA (cfDNA), and extracellular vesicles (EVs). EEBC are exfoliated tumor cells present in urine that need to be enriched to improve the sensitivity of the assay. Early methods used direct isolation from urine samples using filter membranes (Croft and Nelson, 1979). Although the sensitivity of a single physical filtration modality in the early stages is low, it still increases the sensitivity in early bladder cancer detection from 80% to 87% (Andersson et al., 2014). In recent years, antibody-based immunological methods have improved, and studies have shown that it is possible to capture scattered cancer cells with up to 99% selectivity and 100% sensitivity, thus achieving significant advances (Macgregor-Ramiasa et al., 2017). However, there are limitations, such as quantity bias caused by EEBC morphology, that need to be solved before this technology can be applied clinically.

After the release of cfDNA from cells during tumor cell necrosis or apoptosis, its DNA molecules cross the renal barrier after entering the circulatory system and are subsequently detected in urine samples (Botezatu et al., 2000; Hentschel et al., 2021). Similarly, cfDNA in urine samples can be isolated and detected in urine through centrifugation or a size-based selection method. The reduction in target DNA caused by, for example, high cfDNA evasion capture is more easily compensated by expanding the volume of the collected sample than for blood samples. Studies have shown that the concentration of cfDNA in urine may be e higher than that in blood (Hirotsu et al., 2019). For example, Hirotsu et al. assessed liquid biopsies and found 168 somatic mutations. These mutations were identified in 53% of the urine supernatants, 48% of the urine sediments, and only 2% of the plasma they assessed.

EVs can be detected in various types of body fluids, including urine. Analysis of circRNA (He et al., 2021) and mRNA (Murakami et al., 2018) contained in EVs revealed genes related to biomarkers, such as SLC2A1, GPRC5A, and KRT17, which are relatively promising in the early detection of diseases (Murakami et al., 2018). This demonstrates their potential as biomarkers for bladder cancer. However, the sample quality and quantity of these studies suggest that further research is required to fully elucidate their potential. EVs have been established as biomarkers for the detection of prostate cancer. The sensitivity and specificity of the EV-derived gene TMPRSS2-ERG used in the diagnosis of prostate cancer exceed 80%, indicating that prostate cancer can be detected without imaging, by using this liquid biopsy (Motamedinia et al., 2016). Clinical studies have also shown that liquid biopsies are highly accurate and play an important role in eliminating unnecessary tissue biopsies, and false negatives (Vlaeminck-Guillem, 2021).

## Discussion

Early detection of solid tumors is important to address the impact of cancer on public health. This article discusses the latest advances and methods in tumor detection. The use of these assays is advantageous; however, they have limitations that affect the accuracy, sensitivity, and specificity with which they detect tumors. Even though research on traditional cancer detection methods still needs to be improved, with the development of new assays, biomarkers used in tumor detection has been identified. Subsequently, there is a trend in combing multiple markers and methods to improve the precision and accuracy of tumor detection. The use of multiple assays to detect multiple biomarkers will need to be further assessed. Reducing the number of manual steps and embracing automation is the ultimate goal in tumor detection. Automating tumor detection will alleviate the public health burden of cancer by making the early detection of cancer inexpensive, accurate, efficient, and fast.
